# 133. Ceftriaxone-resistant Haemophilus influenzae in Korean children

**DOI:** 10.1093/ofid/ofac492.211

**Published:** 2022-12-15

**Authors:** Sanghoon Lee, Euntaek Lee, Gahee Kim, Jung Hwa Kim, Mina Kim, Ji-Na Lee

**Affiliations:** Asan medical center, Seoul, Seoul-t'ukpyolsi, Republic of Korea; Asan medical center, Seoul, Seoul-t'ukpyolsi, Republic of Korea; Asan Medical Center, University of Ulsan College of Medicine, Seoul, Kyonggi-do, Republic of Korea; Asan medical center, Seoul, Seoul-t'ukpyolsi, Republic of Korea; Asan medical center, Seoul, Seoul-t'ukpyolsi, Republic of Korea; Asan medical center, Seoul, Seoul-t'ukpyolsi, Republic of Korea

## Abstract

**Background:**

*Haemophilus influenzae* is known to develop resistance to β-lactam antibiotics by either producing β-lactamase or modifying structure of penicillin-binding protein 3 (PBP3) by *ftsI* gene mutation. Since 2017, *H. influenzae* with ceftriaxone resistance has been continuously appearing in our hospital, and the genotypic characteristics of these strains was analyzed.

**Methods:**

Among a total of 69 *H. influenzae* isolated from the patients who admitted to Asan Medical Center Children’s Hospital from March 2014 to April 2019, 23 isolates resistant to ceftriaxone by EUCAST disk diffusion method were included. The ceftriaxone MIC was checked again with the E-test and ceftriaxone MIC ≤ 0.125 mg/L were categorized as susceptible. As test for β-lactamase production, the cefinase test and PCR amplification for *bla*_TEM-1_ and *bla*_ROB-1_ were performed. The sequencing of the *ftsI* encoding PBP3 amplified by PCR was performed, and amino acid sequences were compared with *H. influenzae* Rd KW20.

**Results:**

Among total 23 ceftriaxone-resistant isolates by disk diffusion method, 21 isolates restored for further molecular characterization were included, and 19% (4/21) were reported as susceptible by E-test. Among the 21 ceftriaxone-resistant isolates, 76.2% (16/21) were non-susceptible to amoxicillin/clavulanate, and one of them were resistant to meropenem. The prevalence of b-lactamase-producers was 57.1% (12/21), all of which were positive for *bla*_TEM-1._ For *ftsI* gene sequencing, either R517H or N526K amino acid substitution was observed, and none had both. The number of isolates having S385T was 17, of which 15 isolates accompanied with both M377I and L389F. The median ceftriaxone MIC of 3 isolates with only N526K was 0.012 ug/ml (range 0.008-0.125 ug/ml), and 12 isolates with N526K, M377I, S385T, and L389F was 0.19 ug/ml (0.19-0.25 ug/ml). Lastly, the three isolates with R517H, M377I, S385T, and L389F showed the highest median ceftriaxone MIC with 0.25 ug/ml (0.19-0.75 ug/ml).

**Conclusion:**

Ceftriaxone-resistant *H. influenzae* isolates in Korea seems to be related to the combination of R517H, M377I, S385T, and L389F by *ftsI* gene mutation. Although clinical concerns about treatment failure may be low, continuous monitoring of the emergence of highly resistant strains is necessary.

**Disclosures:**

**All Authors**: No reported disclosures.

